# Characterization of Milkisin, a Novel Lipopeptide With Antimicrobial Properties Produced By *Pseudomonas* sp. UCMA 17988 Isolated From Bovine Raw Milk

**DOI:** 10.3389/fmicb.2018.01030

**Published:** 2018-05-28

**Authors:** Margot Schlusselhuber, Justine Godard, Muriel Sebban, Benoit Bernay, David Garon, Virginie Seguin, Hassan Oulyadi, Nathalie Desmasures

**Affiliations:** ^1^UNICAEN, UNIROUEN, ABTE, Normandie Université, Caen, France; ^2^UNIROUEN, INSA Rouen, CNRS, COBRA, Normandie Université, Rouen, France; ^3^UNICAEN, SF ICORE 4206, Normandie Université, Caen, France

**Keywords:** antimicrobial activity, *Pseudomonas*, milkisin, amphisin, lipopeptide

## Abstract

Biosurfactants such as lipopeptides are amphiphilic compounds produced by microorganisms such as bacteria of the genera of *Pseudomonas* and *Bacillus*. Some of these molecules proved to have interesting antimicrobial, antiviral, insecticide and/or tensio-active properties that are potentially useful for the agricultural, chemical, food, and pharmaceutical industries. Raw milk provides a physicochemical environment that is favorable to the multiplication of a broad spectrum of microorganisms. Among them, psychrotrophic bacterial species, especially members of the genus *Pseudomonas*, are predominant and colonize milk during cold storage and/or processing. We isolated the strain *Pseudomonas* sp. UCMA 17988 from raw cow milk, with antagonistic activity against *Listeria monocytogenes*, *Staphylococcus aureus*, and *Salmonella enterica* Newport. Antimicrobial molecules involved in the antagonistic activity of this strain were characterized. A mass spectrometry analysis highlighted the presence of four lipopeptides isoforms. The major isoform (1409 *m*/*z*), composed of 10 carbons in the lipidic chain, was named milkisin C. The three other isoforms detected at 1381, 1395, and 1423 *m*/*z*, that are concomitantly produced, were named milkisin A, B and D, respectively. The structure of milkisin, as confirmed by NMR analyses, is closely related to amphisin family. Indeed, the peptidic chain was composed of 11 amino acids, 9 of which are conserved among the family. In conclusion, *Pseudomonas* sp. UCMA 17988 produces new members of the amphisin family which are responsible for the antagonistic activity of this strain.

## Introduction

Biosurfactants are amphiphilic compounds produced by microorganisms such as bacteria, yeasts, and some filamentous fungi (Santos et al., 2016). They contain hydrophobic and hydrophilic groups that confer the ability to accumulate between fluid phases, thereby reducing surface and interfacial tension at the surface and interfacial regions, respectively (Kapadia and Yagnik, 2013). Among these molecules, some of them were shown to have interesting antimicrobial, antiviral, insecticide, and/or tensioactive properties that are potentially useful for the agricultural, chemical, food, and pharmaceutical industries (Inès and Dhouha, 2015).

Biosurfactants, such as lipopeptides and glycolipids, are in most cases low-molecular mass membrane-active compounds (usually ranging from 500 to 1500 Da) (Janek et al., 2010). They are mainly produced by bacteria of the genera *Pseudomonas* and *Bacillus* with 70 and 90 different lipopeptides produced, respectively (Santos et al., 2016; Coutte et al., 2017). Recently, a unique online database dedicated to non-ribosomal peptides has been opened (Flissi et al., 2016). 139 lipopeptides produced by *Pseudomonas* have been recorded and classified into 15 family based on their structures. The better known being amphisin, pyoverdine, viscosin, tolaasin, and syringopeptin groups^1^.

Several isoforms can be produced by one single strain which differs either by the length of the fatty acid chain or by the composition of the peptide moiety. In *Pseudomonas* and *Bacillus* genera, most lipopeptides are synthesized in a ribosome-independent manner with mega enzymes called non-ribosomal peptide synthetases (NRPSs) (Inès and Dhouha, 2015). Those synthetases possess a multimodular structure in which each module is involved in the stepwise incorporation of an amino acid in the lipopeptide peptide moiety. A typical module can be subdivided into initiation and elongation modules. A typical initiation module consists of an adenylation (A) domain, responsible for amino acid selection and activation, a thiolation (T or alternatively PCP) domain responsible for thioesterification of the activated amino acid and a condensation (C) domain. The C domain of the first module catalyzes *N*-acylation of the first amino acid, thereby linking the lipid moiety to the oligopeptide. Elongation modules contain A, T, and C domains in which the C domain is responsible for peptide bond formation between two neighboring substrates to elongate the peptide chain. These catalytic domains generate a linear peptide which is cleaved at the end of the assembly line by a thioesterase (TE domain), which results in the release of a linear product or a cyclic compound via an intramolecular cyclization reaction. Additional domains may include an epimerization domain, responsible for the conversion of the L- or D-configuration of an amino acid (Raaijmakers et al., 2010; Roongsawang et al., 2010).

Raw milk provides a physicochemical environment that is favorable to the multiplication of a broad spectrum of microorganisms. Indeed, more than 100 genera and 400 microbial species have been detected in raw milk (Montel et al., 2014). Among them, psychrotrophic bacterial species, especially members of the genus *Pseudomonas*, are predominant and contaminate milk at different steps from farm to dairies (Mallet et al., 2012), and develop during cold storage and/or processing (De Jonghe et al., 2011). *Pseudomonas* sp. are aerobic, Gram-negative bacteria and many of them are able to produce heat-stable extracellular enzymes (Herrera, 2000). Because of their ability to produce proteases, able to degrade casein, and lipase, causing triglycerides degradation, pseudomonads may cause spoilage in dairy products (Cousin et al., 2001). Within this genus, the *P. fluorescens* group is generally known to frequently cause spoilage of stored milk (Arslan et al., 2011) and of some cheese varieties, although it has also been described on the surface of unspoiled cheese (Larpin-Laborde et al., 2011; Wolfe et al., 2014).

In this work, we aim to describe a novel lipopeptide produced by an isolated strain from bovine raw milk collected in Normandy (France) and identified as *Pseudomonas* sp. UCMA 17988. This strain produces a novel antibacterial cyclic lipopeptide along with three isoforms supposed to belong to the amphisin family. The structure of the newly discovered biosurfactant, named milkisin, isolated by RP-HPLC, was determined by mass spectrometry (MALDI TOF) and nuclear magnetic resonance (NMR).

## Materials and Methods

### Isolation and Antibacterial Activity of *Pseudomonas* sp. UCMA 17988

Raw bulk tank milk samples collected on farm in Normandy (France) were screened for isolates with antibacterial properties by a double layer agar method. After 10-fold dilution of the samples, 100 μl of each dilution was spread over the surface of brain heart infusion (BHI) agar plates supplemented with 0.2% glucose and incubated overnight at 30°C. After incubation, 8 ml of BHI soft agar supplemented with 0.2% glucose was mixed with 1% (vol/vol) fresh inoculum prepared from overnight culture of bacterial pathogens and overlaid on agar plates containing less than 100 colonies. The bacterial targets used for the screening process were *Listeria monocytogenes* WSLC 1685, *Staphylococcus aureus* CIP 53.154, *Salmonella enterica* serotype Newport CIP 105629, and *Escherichia coli* O157:H7 *stx*-C267 (Vernozy-Rozand et al., 2000). The plates were then incubated overnight at 30°C. Milk isolates with antagonistic activity were detected by observation of a zone of inhibition around the colonies. The antagonistic *Pseudomonas* sp. UCMA 17988, detected by this method, was picked up with sterile toothpick for purification by isolation using Tryptone Soya Agar – Yeast Extract (TSA-YE) agar plates.

The antagonistic properties of *Pseudomonas* sp. UCMA 17988 were then studied using the spot-on-lawn technique against selected bacterial strains: *L. monocytogenes* WSLC 1685, *S. aureus* CIP 53.154, *E. coli* O157:H7 *stx*-C267, and *S. enterica* serotype Newport CIP 105629, serotype Typhimurium LMG 7233, serotype Dublin CIP 7053, serotype Mbandaka CIP 105859, and serotype Montevideo CIP 104583. To do this, soft BHI agar plates supplemented with 0.2% glucose were inoculated with target strains. Inoculation was performed with cotton swab dipped into adjusted inoculum (OD_600_ 0.01). Excess liquid was removed by gentle rotation of the cotton swab against the inner surface of the test tube. To obtain even growth, the entire agar surface was swabbed uniformly by the cotton swab. The inoculated plates were left at room temperature for 3–5 min to allow any surface moisture to be absorbed. 2.5 μl of *Pseudomonas* sp. UCMA 17988 overnight culture was then spotted prior to incubation for 24 h at 30°C. Subsequently, the clear zone around the spot was recorded.

### PCR Amplification and DNA Sequencing Conditions

Colony PCR amplification and DNA sequencing of *rpoD*, and *16S rRNA* genes were performed for identification of *Pseudomonas* sp. UCMA 17988 strain. The *16S rRNA* gene sequence was amplified using the primers W18F (5′-GAGTTTGATCMTGGCTCAG-3′) and W02R (5′-GNTACCTTGTTACGACTT-3′) respecting the following PCR conditions: initial hot-start step at 98°C for 10 min, 35 cycles of denaturation at 98°C for 10 s, annealing at 49.5°C for 20 s, and extension at 72°C for 45 s; and a final extension of 10 min at 72°C. *rpoD* gene was amplified using the primers set (PsEG30F/PsEG790R), as described by Mulet et al. (2010), respecting the following PCR conditions : 5 min 98°C, 30 cycles (98°C 10 s, 55°C 30 s, 72°C 25 s) 5 min 72°C. All PCR reactions were conducted with Thermo Scientific Phusion High-Fidelity PCR Master Mix prior sequencing by GATC Biotech (Cologne, Germany), with Sanger method. Forward and reverse strands were assembled with Bioedit software and deposited to GenBank. The sequence was then compared to the GenBank database (NCBI).

### Phylogenetic Analyses

Phylogenetic analyses were carried out using *rpoD* and *16S rRNA* gene sequences of *Pseudomonas* sp. UCMA 17988 with type strains obtained from public database (von Neubeck et al., 2017). Alignments were performed by using Muscle Alignment tool (Edgar, 2004) with default settings and were used for the calculation of maximum-likelihood phylogenetic trees based on the Tamura–Nei model. All methods used are embedded in the MEGA 7 software. The statistical evaluation of the tree topologies was performed by bootstrap analysis with 1000 resamplings.

### Culture Conditions and Biosurfactants Extraction

The strain was routinely cultured on TSA-YE (6 g/l) at 30°C. For biosurfactants production, the strain was grown in flasks containing 200 ml mineral salt medium (MSM) with the following composition: 7 g/l K_2_HPO_4_, 2 g/l KH_2_PO_4_, 1 g/l (NH_4_)_2_SO_4_, 0.5 g/l sodium citrate 2H_2_O, and 0.1 g/l MgSO_4_ × 7H_2_O (pH 7.0). Glucose was added to MSM to a final concentration of 20 g/l (Janek et al., 2010). The flasks were inoculated with overnight liquid culture at a final OD_600_ = 0.01 and then incubated at 17°C for 4 days under agitation (180 rpm; INFORS Unitron). After incubation, bacteria were removed by centrifugation at 10,000 × *g*, for 30 min at 4°C. The culture supernatant was filtered against a 0.22-μm filter.

The supernatant was subjected to drop-collapse assay for rapid screening of biosurfactants production. Briefly, 5 μl of supernatant was placed on an oil-coated surface with 1 μl of methylene blue to improve visualization. The stability of the drop was checked and compared to a negative control (sterile MSM medium). If the liquid contains surfactants, the drops spread because of the reduction of the force or interfacial tension between the liquid drop and the hydrophobic surface (Walter et al., 2010).

A cell-free supernatant was extracted three times with ethyl acetate (1:1, v/v). The organic fractions were collected and evaporated under vacuum. The residual crude extract was dissolved in water/acetonitrile (ACN) (1:1, v/v) and stored at 4°C until analysis.

### Time Course of Cell Growth and Lipopeptide Production

*Pseudomonas* sp. UCMA 17988 was grown in liquid MSM medium containing 20 g/l of glucose. Flasks were inoculated with overnight liquid culture at a final OD_600_ = 0.01 and then incubated at 17°C for 6 days under agitation (180 rpm; INFORS Unitron). The growth was evaluated every 24 h by measuring optical density at 600 nm. The production of lipopeptide was measured by HPLC using a previously reported method (Luo et al., 2013).

### Antibacterial Effect of Crude Extract by Well-Diffusion Method

The antibacterial effect of crude extract was analyzed using the well-diffusion technique (Tagg and McGiven, 1971) modified by Audisio et al. (2005). The lawn was obtained by sowing 100 μl of a 24-h indicator strain (*L. monocytogenes* WSLC 1685, *S. aureus* CIP 53.154, and *S. enterica* serotype Newport CIP 105629) culture in 20 ml of TSA-YE soft agar (0.75%, w/v). Fifty microliters of crude extract was poured into wells punched in the lawn of the indicator strain and the plates were incubated at 30°C for 24 h. Fifty microliters of diluent (water/ACN, 1:1) was used as negative control. After incubation, plates were examined for the presence of inhibition halos.

### HPLC and Mass Spectrometry

Reverse phased HPLC (Waters Alliance HPLC system with 2695 pump and 2998 PDA detector) was performed at room temperature at 1 ml/min on a C18 Chromolith SpeedROD RP-18e column (4.6 × 50 mm, Merck Millipore, United States). Mobile phase: H_2_O (0.1% formic acid)/ACN; 0 min: 50/50, 2 min: 50/50, 22 min: 0/100, 25min: 0/100. The elution was monitored at 220 nm. Fractions were collected and lyophilized prior storage at -20°C.

Mass spectrometry analyses were carried out on a MALDI AB Sciex 5800 proteomics analyzer equipped with TOF ion optics and an OptiBeam on-axis laser irradiation with 1000 Hz repetition rate. The system was calibrated before analysis with a mixture of Angiotensin I, Angiotensin II, Neurotensin, ACTH clip (1–17), ACTH clip (18–39), and mass precision were better than 50 ppm. A 1 μl volume of biosurfactant solution was mixed with 10 μl of alpha-cyano-4-hydroxy-cinnamic-acid (CHCA) matrix prepared in a diluent solution of 50% ACN with 0.1% trifluoroacetic acid. The mixture was spotted on a stainless steel Opti-TOF 384 targets; the droplet was allowed to evaporate before introducing the target into the mass spectrometer. Acquisitions were taken in automatic mode. A laser intensity of 3400 was typically employed for ionizing. MS spectra were acquired in the positive reflector mode by summarizing 1000 single spectra (5 × 200) in the mass range from 700 to 2000 Da. MS/MS spectra were acquired in positive MS/MS reflector mode by summarizing a maximum of 2500 single spectra (10 × 250) with a laser intensity of 3900. For the tandem MS experiments, the acceleration voltage applied was 1 kV and air was used as the collision gas. Gas pressure medium was selected as settings. The fragmentation pattern was used to determine the sequence of the lipopeptide.

### NMR Spectroscopy

Nuclear magnetic resonance spectra were obtained using a Brüker AVIII 600 spectrometer (Brüker, Wissembourg, France) equipped with a 10 Å gradient amplifier and a 5 mm CPTX {^1^H, ^13^C, ^15^N} including shielded *z* gradients. A lyophilized sample was dissolved in acetone-d6. The 1D ^1^H, 2D ^1^H–^1^H (COSY, TOCSY, NOESY) and ^1^H–^13^C (HSQC, HMBC) spectra were recorded at 298 K.

### Inhibition Spectrum of Milkisin

The minimum inhibitory concentrations (MICs) of purified lipopeptide were evaluated in triplicate by using a microtiter plate dilution assay as outlined by the Clinical & Laboratory Standards Institute in the CLSI M07-A10 document (CLSI, 2015). Target bacterial strains *S. aureus* CIP 53.154, *S. enterica* Newport CIP 105629, *S. enterica* Typhimurium LMG 7233, *S. enterica* Mbandaka CIP 105859, *S. enterica* Dublin CIP 7053, *S. enterica* Montevideo CIP 104583, *L. monocytogenes* WSLC 1685, *E. coli* O157:H7 *stx*-C267S, *E. coli* K12 ATCC 1079, *Enterococcus faecium* CIP 103014T, and *Pseudomonas aeruginosa* LMG 01242T were grown overnight and adjusted to 5.10^5^ CFU/ml in Müller Hinton broth (MHB). Lipopeptide concentrations were tested up to 1 mg/ml. The microtiter plates were incubated at 30°C until visible growth of the positive control. Cultures without peptides were used as positive controls. Non-inoculated MHB was used as a negative control. MICs were defined as the lowest concentration of an antimicrobial that inhibits the visible growth of a microorganism after overnight incubation.

Four fungal strains were also chosen to test different representative fungal groups: *Aspergillus niger* (CMPG 814), *Cladosporium herbarum* (CMPG 38), *Mucor hiemalis* (CBS 201.65), and *Penicillium expansum* (CMPG 136). The fungal stock cultures were incubated for 15 days at 25°C on malt extract agar (MEA) medium. Each culture was scrapped to recover mycelium and spores which were crushed in sterile water (Ultra Turax^®^, United States) and filtered on a sterile gauze in order to obtain a fungal suspension. Then 1 ml of the final inoculum calibrated at 10^6^ spores/ml was deposited on Petri dish. The solution of milkisin (20 mg/l) and the four dilutions (2, 1, 0.5, and 0.25 mg/ml) were deposited in triplicate on sterile filter paper discs (6 mm in diameter) placed on the agar surface of Petri dishes according to the agar disc diffusion method (Balouiri et al., 2016). The growth inhibition around the disc was observed after both 48 and 72 h. The sensitivities of the fungal species to the milkisin were determined by measuring the sizes of inhibitory zones (including the diameter of the disc). Tests were performed in triplicate.

## Results

### Properties of *Pseudomonas* sp. UCMA 17988

Comparison of the *16S rRNA* gene sequence (1398 nt, Accession Number: MH016575) with type strains of bacterial species recorded in the GenBank database assigned the strain to the *Pseudomonas* genus. The closest relative was *Pseudomonas helmanticensis* OHA11^T^ with 99% pairwise identities (5 nt differences). The next closest relatives with 99% identity (6–16 nt differences) were the type strains *P. baetica* a390^T^, *P. umsongensis* Ps3-10, *P. jessenii* CIP 105274, *P. synxantha* LMG 2190, *P. granadensis* LMG 27940, *P. reinekei* MT1, *P. koreensis* Ps 9-14, *P. mohnii* lpA-2, *P. vancouverensis* DhA-51, *P. granadensis* F-278,770^T^, and *P. mandelii* CIP 105273. A phylogenetic analysis of *16S rRNA* gene sequences was carried out including all the closest related species to *Pseudomonas* sp. UCMA 17988. According to the maximum-likelihood phylogenetic tree, the strain clustered in a separate branch related to *P. helmanticensis*, *P. baetica*, *P. reinekei*, *P. umsongensis*, *P. mohnii*, *P. jessenii*, and *P. vancouverensis* (data not shown).

**FIGURE 1:**
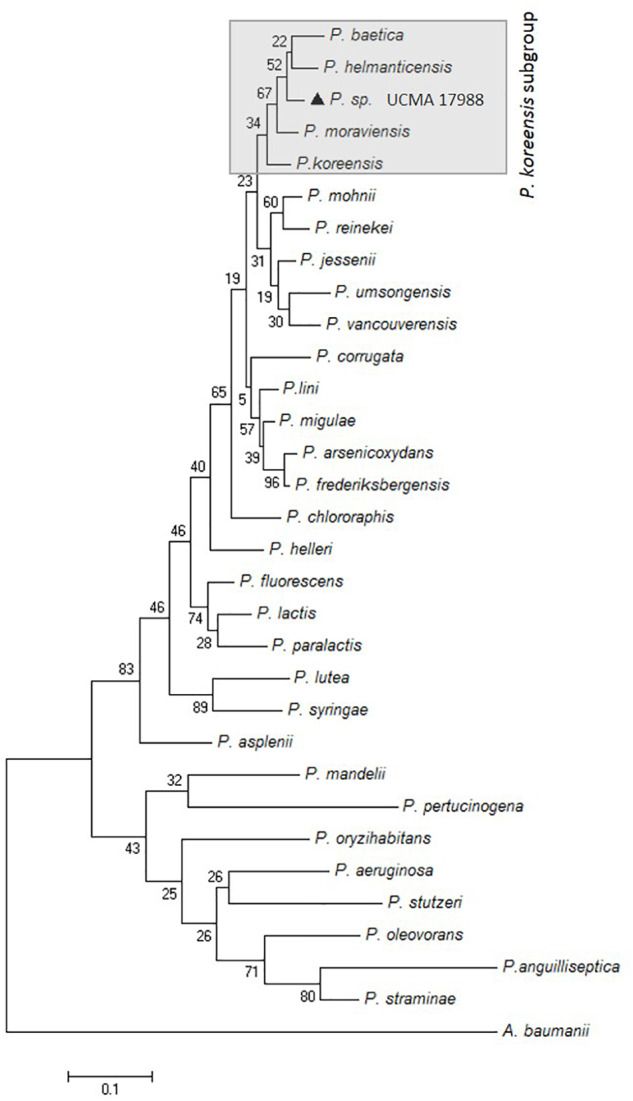
Maximum-likelihood phylogenetic tree based on partial *rpoD* gene sequences (727 nt) of *Pseudomonas* sp. UCMA 17988 (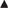) and closely related species. The percentage of replicate trees in which the associated taxa clustered together in the bootstrap test (1000 replicates) is shown next to the branches. *Acinetobacter baumannii* was used as an outgroup.

The resolution of *16S rRNA* gene sequences at the intrageneric level being low, the gene sequence for RpoD, providing better resolution, has been studied (Mulet et al., 2010; Rajwar and Sahgal, 2016). Indeed, among *Pseudomonas* genus, the housekeeping *rpoD* gene possesses the highest discriminatory power when compared to other useful genes to analyze phylogenetic relationships among the genera (e.g., *rpoB*, *gyrB*, *16S rRNA* gene sequences) (Mulet et al., 2010). The comparison of partial *rpoD* nucleotide sequences revealed the highest pairwise identity to *P. helmanticensis* OHA11^T^ (95%; 35 nt differences) and about 94% with *P. granadensis* LMG 27940, *P. granadensis* F-278,770^T^, and *P. moraviensis* DSM 16007^T^. The nucleotide sequence of *rpoD* gene of the strain reported here (698 nt, accession number: MH094851) clustered in *P. koreensis* subgroup (**Figure 1**).

**FIGURE 2:**
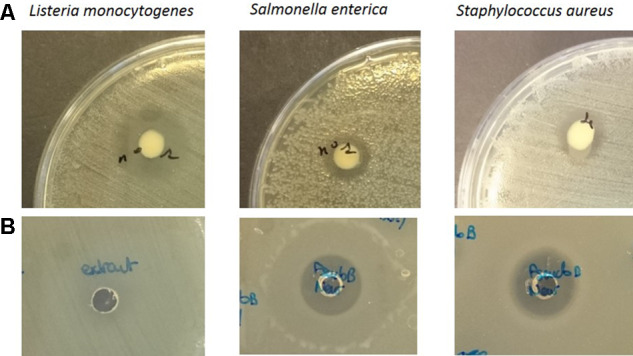
Spot on the lawn assay of *Pseudomonas* sp. UCMA 17988 **(A)** and well diffusion assay of biosurfactants crude extract **(B)** against *Listeria monocytogenes* WSLC 1685, *Salmonella enterica* Newport CIP 105629, and *Staphylococcus aureus* CIP 53.154.

*Pseudomonas* sp. UCMA 17988 showed antibacterial activity by the spot-on-lawn method against various Gram-negative and Gram-positive bacterial pathogens. Indeed, inhibition could be observed for *L. monocytogenes* WSLC 1685, *S. aureus* CIP 53.154, and *S. enterica* serotype Newport CIP 105629 (**Figure 2**). Interestingly, no activity could be observed for the other *Salmonella* serotypes tested (Typhimurium, Dublin, Mbandaka, and Montevideo).

### Extraction and Structural Analysis of Biosurfactants

**FIGURE 3:**
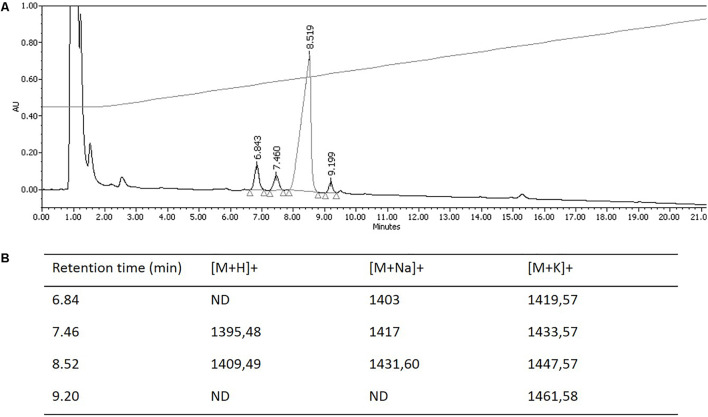
Purification and mass spectrometry analysis of biosurfactants isoforms produced by *Pseudomonas* sp. UCMA 17988. **(A)** Reverse-phase HPLC. Elution was carried out using H2O/ACN/formic acid 0.1% (v/v) solvent system and monitored at 220 nm. **(B)** Pseudo-molecular ions detected by MALDI-TOF analysis. ND, not detected.

The hypothesis that *Pseudomonas* sp. UCMA 17988 produces biosurfactants was explored in order to explain the antimicrobial activity of the strain. After a 4-day bacterial growth in MSM medium with glucose as sole carbon source, the highly reduced surface tension of the cell-free supernatant indicated the production of biosurfactants by the strain (Supplementary Figure S1). Biosurfactants produced were isolated using a two-step purification method. First, biosurfactants were partially purified from the culture supernatant by ethyl acetate extraction. After the first step of purification, the antibacterial activity of the extract was checked using the well-diffusion method (**Figure 2**). Biosurfactants were further purified from the extract using reverse phase HPLC on a C18 column. HPLC chromatogram highlights four peaks, with retention time at 6.84, 7.46, 8.52, and 9.20 min (**Figure 3**).

**FIGURE 4:**
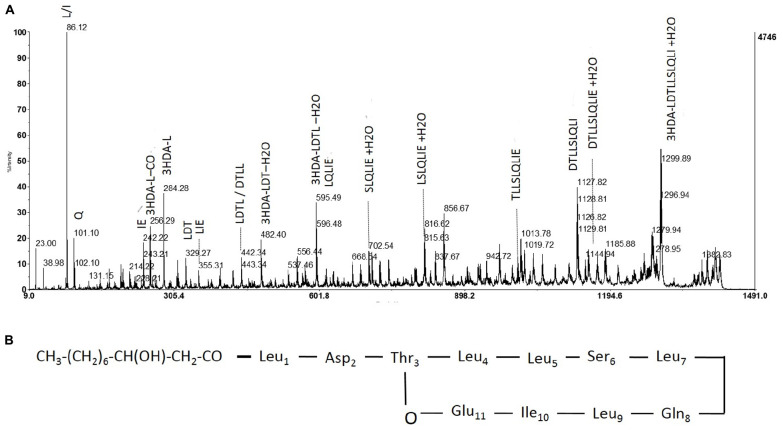
Fragmentation pattern of lipopeptide *m*/*z* 1409 and proposed structure. **(A)** Product ions obtained by fragmentation using MALDI-TOF. **(B)** Determined structure of purified lipopeptide (*m*/*z* 1409) based on mass spectrometric and NMR analysis.

**FIGURE 5:**
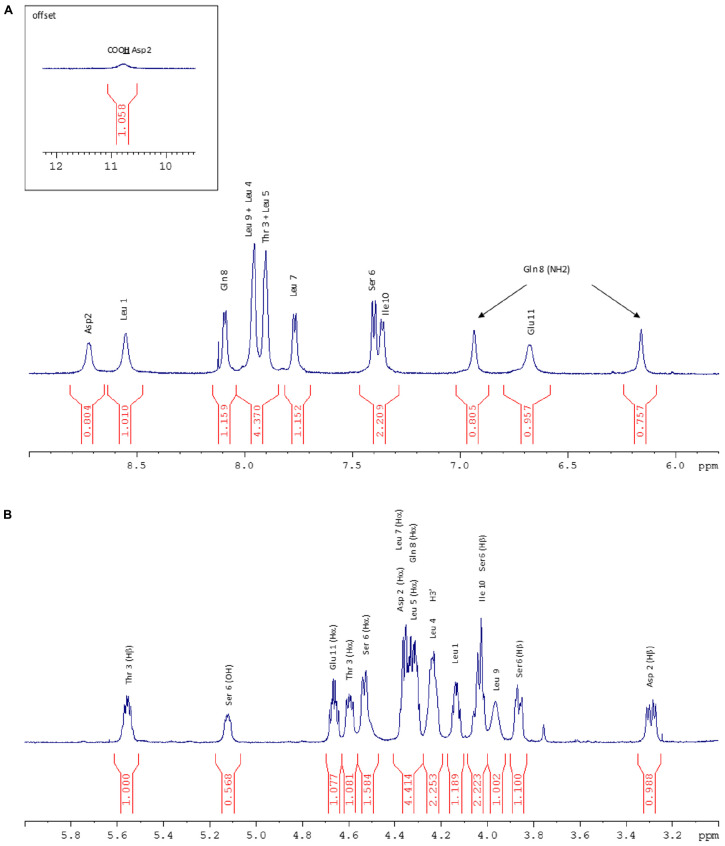
**(A)** NH zone of the ^1^H NMR spectrum (600 MHz, 298 K, Acetone-d_6_). **(B)** Hα zone of the ^1^H NMR spectrum (600 MHz, 298 K, Acetone-d_6_).

Hereafter, collected fractions were analyzed by MALDI-TOF mass spectrometry. The spectra also highlighted the presence of four molecules (**Figure 4**). Two intense pseudomolecular ions [M+H]+ at *m*/*z* 1409.49 and 1395.48 were observed. Corresponding sodium and potassium adducts were also found at *m*/*z* [M+Na]+ at 1417 and 1431.60 and *m*/*z* [M+K]+ at 1433.57 and 1447.57 respectively. The two other molecules could be detected through their potassium and/or sodium adducts (*m*/*z* [M+Na]+ at 1403; *m*/*z* [M+K]+ at 1419.57 and 1461.58). The presence of four molecules harboring a difference of 14 Da was consistent with the presence of lipopeptides isoforms. Indeed, such difference is typical of the addition or substitution of a methyl group in the fatty acid chain. A MS/MS analysis was performed on the dominant [M+H]+ ion (*m*/*z* at 1409.49). The fragmentation spectrum obtained is shown in **Figure 5**. Fragments were assigned by subtraction of the different peaks between them. The ions with 284 Da and 86 Da masses were observed and respectively correspond to fragment 3-hydroxy-fatty acid-Leu/Ile and immonium ion of leucine or isoleucine. The following linear sequence was proposed: 3HDA-Leu/Ile1-Asp2-Thr3-Leu/Ile4-Leu/Ile5-Ser6-Leu/Ile7-Gln8-Leu/Ile9-Leu/Ile10-Glu11 with cyclisation between Thr3 and Glu11.

In order to confirm the isoforms hypothesis, three other molecules underwent a fragmentation process. Indeed, the MS/MS results confirmed the difference of 14 Da only in fragments carrying the fatty acid chain indicating the presence of four isoforms of the same lipopeptide (Supplementary Figures S2–S4). In order to confirm the lipopeptide sequence, especially because of the high proportion of leucine and/or isoleucine in the peptidic moiety, an NMR analysis was performed (Supplementary Table S1).

1D and 2D ^1^H and ^13^C NMR spectra contained predominantly resonances that were characteristic of peptides. In the low field region (6–11 ppm) of the 1D ^1^H NMR spectrum (**Figure 5A**), a broad signal at 10.78 ppm corresponding to an acidic proton (Asp2), signals of 11 NH protons and a pair of signals characteristic of NH2 protons were observed. Between 1 and 6 ppm (**Figure 5B**), the characteristic signals of the aliphatic CH (α, β, γ, and δ) protons appeared with residual solvents (water and acetone). Finally, between 0.8 and 1 ppm some signals corresponding to 45 protons that is to say 15 methyl groups were observed.

**FIGURE 6:**
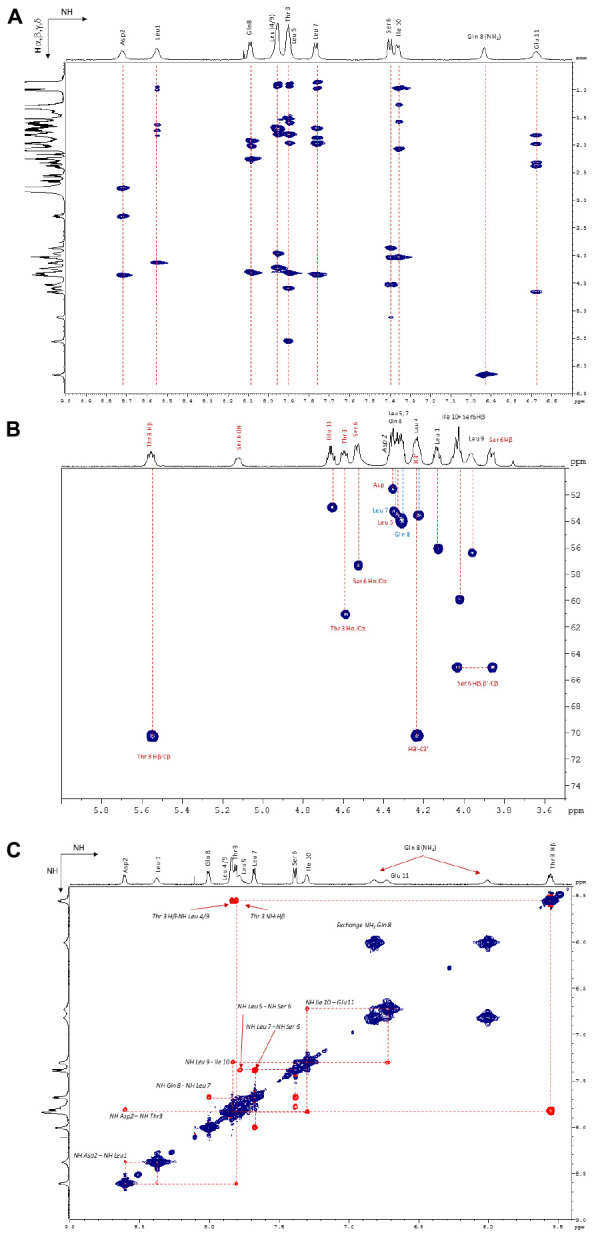
**(A)** NH/Hα, β, and γ zone of the ^1^H–^1^H TOCSY NMR spectrum (600 MHz, 298 K, Acetone-d_6_). **(B)** Hα/Cα zone of the ^1^H–^13^C HSQC NMR spectrum (600 MHz, 298 K, Acetone-d_6_). **(C)** NH/NH zone of the ^1^H–^1^H NOESY NMR spectrum (600 MHz, 323 K, Acetone-d_6_).

The ^1^H NMR spectrum was assigned by the standard procedure (Wüthrich, 1986). The first step involved the analysis of COSY, TOCSY (**Figure 6A**), and NOESY (**Figure 6B**) spectra to identify spin systems that are characteristic of particular amino acids. The COSY spectrum showed correlations HNi-HαI for each residue which excluded the presence of a proline in the lipopeptide. Then the spin system residues were assigned to specific locations in their sequence by the observation of NOE between resonances of sequentially adjacent residues, HNi-HNi+1, Hαi-HNi+1, and Hβi-HNi+1. The NH proton of Leu1, which is the only Leu out of the macrocycle, was identified in accordance with the literature (Weisshoff et al., 2014) and from a NOE correlation with H2’ (2.55 ppm) as the most deshielded Leu NH (8.55 ppm) and then served as a sequential starting point. The observation of NOES due to HNi-HNi+1 and Hαi-HNi+1 allowed assignment of fragments placed in a unique position in the sequence. The NH proton of Leu1 is weakly correlated with NH proton of Asp residue which is correlated to Thr3 NH suggesting Asp residue located in position 2. Once the assignment of the protons of each residue was realized, the ^1^H–^13^C HSQC and the ^1^H–^13^C HMBC spectra allowed the assignment of the corresponding protonated and quaternary carbons. The 2D ^1^H–^13^C HSQC spectrum (**Figure 6C**) pointed out two carbinol methine carbons (CHOH) at 70.33 ppm corresponding to the Cβ of Thr3 (attached to Thr3 Hβ at 5.56 ppm) and to the C3’ (attached to H3’ at 4.24 ppm) of the fatty chain. The downfield chemical shift of Thr3 Hβ (5.56 ppm) indicated that the hydroxyl group was part of an ester linkage implying that the cyclization involved this residue. All ^1^H and ^13^C resonance assignments are summarized in **Table 1** (Supporting Information).

**Table 1:** Antimicrobial activity of milkisin.

Microorganism	MIC (mg/ml)
*Staphylococcus aureus* CIP 53.154	0.5
*Salmonella enterica* serotype Newport CIP 105629	1
*Salmonella enterica* serotype Typhimurium LMG 7233	>1
*Salmonella enterica* serotype Dublin CIP 7053	>1
*Salmonella enterica* serotype Mbandaka CIP 105859	>1
*Salmonella enterica* serotype Montevideo CIP 104583	>1
*Escherichia coli* O157:H7 *stx*-C267S	>1
*Listeria monocytogenes* WSLC 1685	>1
*Pseudomonas aeruginosa* LMG 01242T	>1
*Escherichia coli* K12 ATCC 1079	>1
*Enterococcus faecium* CIP 103014T	>1
*Aspergillus niger* CMPG 814	>20
*Cladosporium herbarum* CMPG 38	>20
*Mucor hiemalis* CBS 201.65	>20
*Penicillium expansum* CMPG 136	20


*Antibacterial activity was determined by broth microdilution (maximum concentration tested: 1 mg/ml). Antifungal activity was determined by agar diffusion test (maximum concentration tested: 20 mg/ml).*

Consequently, ^1^H, ^13^C NMR, as well as MSMS data confirm the sequence represented in **Figure 4**.

### Time Course of Cell Growth and Lipopeptide Production

**FIGURE 7:**
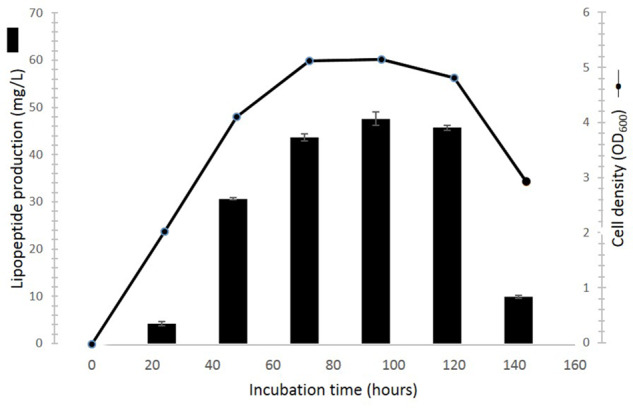
Time course of cell growth and lipopeptides production of *Pseudomonas* sp. UCMA 17988 in glucose-MSM medium. Cell growth was evaluated by optical density measurement at 600 nm. Lipopeptide was quantified by HPLC.

The time course of the cultivation is shown in **Figure 7**. Lipopeptides concentration increased rapidly during the exponential phase of growth thereby suggesting that the molecules are produced as primary metabolites accompanying cellular biomass formation. The average maximum yield of lipopeptides (47.6 ± 1.4 mg/l) was reached at 96 h cultivation. During cell apoptosis period (120–144 h), the lipopeptides production dramatically decreased by 78.6%.

### Antimicrobial Activity of Milkisin

The antibacterial activity of the major isoform was evaluated against 11 Gram-positive and Gram-negative bacterial strains. Antibacterial activity was observed against *S. aureus* CIP 53.154 and *S. enterica* Newport CIP 105629 strains with MIC of 0.5 and 1 mg/ml, respectively. The antifungal activity was also investigated against four strains representative of fungal groups (*P. expansum* CMPG 136, *M. hiemalis* CBS 201.65, *A. niger* CMPG 814, and *C. herbarum* CMPG 38). Only a weak antifungal activity was observed against *P. expansum* CMPG 136 (MIC of 20 mg/l). The antimicrobial spectrum of milkisin is presented in **Table 1**.

## Discussion

**FIGURE 8:**
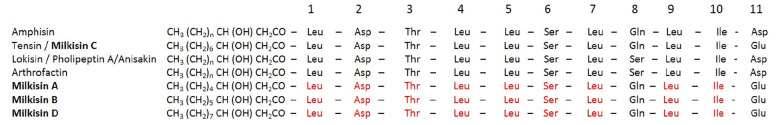
Alignment of lipopeptide milkisin isoforms produced by *Pseudomonas* sp. UCMA 17988 with members of amphisin group.

In the present study, we aim to describe the production of new lipopeptides by *Pseudomonas sp*. UCMA 17988 isolated from raw milk. To our knowledge, the sequence did not correspond to any known lipopeptide in the literature or recorded in the NORINE database. Nevertheless, the sequence was very close to members of the amphisin group since the peptidic chain was composed of 11 amino acids, 6 of which are conserved among the family (**Figure 8**). The name “milkisin,” chosen for this new lipopeptide, refers to the matrix in which *Pseudomonas* sp. UCMA 17988 has been isolated. The major isoform (1409 *m*/*z*), composed of 10 carbons in the lipidic chain, was named milkisin C. The molecule has been recorded in the NORINE database under the identification number NOR01358. The three other isoforms of 1381, 1395, and 1423 *m*/*z*, that are concomitantly produced, were named milkisin A, B, and D, respectively. As far as we know, only two *Pseudomonas* strains isolated from dairy product were shown to produce cyclic lipopeptides. However, those two strains, closely related to *P. tolaasii*, were shown to produce isoforms belonging to another family (viscosin) (Reybroeck et al., 2014).

The present study provides evidence that the production of milkisin is tightly coupled to cell proliferation. This is consistent with the results obtained for other members of the amphisin group, such as tensin and amphisin production for example (Nielsen et al., 2000; Koch et al., 2002). Indeed, the *Pseudomonas* sp. UCMA 17988 starts producing milkisin at the beginning of the exponential phase. Lipopeptides accumulate during the stationary phase before to dramatically decrease along with cell death. These observations are also consistent with the synthesis of other lipopeptides among the *Bacillus* and *Pseudomonas* genera (Saikia et al., 2012; Luo et al., 2013).

In the literature, several lipopeptides have potent antifungal potential and antibacterial activities. However, the antibacterial activity is mostly moderate and confined to Gram-positive bacteria since Gram-negative bacteria seem to be better protected thanks to their different cell envelopes (Reder-Christ et al., 2012). Few studies focusing on the antimicrobial potential of members of the amphisin group composed of anikasin, amphisin, arthrofactin, lokisin, tensin, and pholipeptin were conducted. These studies highlighted the weak antimicrobial potential of this group. For example, in a comparative study, 1 mg/ml of arthrofactin did not show inhibition of a large panel of Gram-positive and Gram-negative bacteria (*E. coli*, *Klebsiella pneumoniae*, *Citrobacter freundii*, *P. aeruginosa*, *Serratia marcescens*, *S. aureus*, *Staphylococcus epidermidis*, *Staphylococcus simulans*, *Micrococcus luteus*, *E. faecium*, *Bacillus subtilis*, *Bacillus megaterium*, *Listeria welshimeri*). A weak activity was only observed against *Corynebacterium* sp., *Mycobacterium smegmatis*, and *Arthrobacter crystallopoietes* (Reder-Christ et al., 2012). The newly discovered anikasin did not show any antibacterial activity at the maximum concentration tested (1 mg/ml) against a variety of different species (*B. subtilis*, *S. aureus*, *E. coli*, *P. aeruginosa*, and *Mycobacterium vaccae*) and was only weakly active against *E. faecium* (Götze et al., 2017). In addition no antifungal properties were observed. Antifungal activity was only reported against *Rhizoctonia solani* with tensin (Nielsen et al., 2000). Consistently with these previous reports, milkisin also only has weak antibacterial properties against the Gram-positive and Gram-negative strains *S. aureus* and *S. enterica* Newport, respectively, and a weak antifungal activity. Surprisingly, no activity could be observed against the four other *Salmonella* strains tested. However, the absence of activity might be attributed to species-specific variations of these strains in the cell-surface or in the cell wall composition. Indeed, it is widely accepted that the primary target of lipopeptides is the membrane. The membrane association is driven both by the polar amino acid residues that initially allow interaction with the polar head groups of the phospholipids and by the following incorporation of the fatty acids into the membrane core. The disintegration of the lipid bilayer causes leakage of ions from the cells(Sinnaeve et al., 2009). The size of the acyl chain appears to be crucial for the antimicrobial activity of lipopeptides, presumably due to a different interaction with the cell membrane. Indeed, few studies revealed a difference in the antimicrobial potency of lipopeptide analogs, the one with longer acyl chain being the most potent (Kracht et al., 1999; Tabbene et al., 2011). Based on these studies, we may speculate that isoforms C and D of milkisin would be more active than shorter isoforms A and B. However, specific activity of each isoform of milkisin could not be analyzed in our study because of the very low amount produced for minor isoforms A, B, and D. More specific mechanisms may also be evolved in some cases, e.g., inhibition of the cell-wall synthesis (Schneider et al., 2009; Reder-Christ et al., 2012). The mode of action of six structurally different *Pseudomonas* lipopeptides, including arthrofactin which is closely related to milkisin, was investigated using a model membrane system (Reder-Christ et al., 2012). The study revealed a high membrane affinity. However, arthrofactin was shown to have a median incorporation tendency and was not able to disrupt the model membrane at 50 μg/ml (Reder-Christ et al., 2012). This may explain the weak antimicrobial activity of both milkisin and arthrofactin.

Until today, lipopeptides have received considerable attention especially for their antifungal and surfactant properties. However, it appears that these molecules have additional natural roles allowing the bacteria to survive in the environment. Indeed, these molecules have been thought to confer competitive advantage in interactions with other organisms such as viruses, mycoplasmas, bacteria, fungi, and oomycetes (Raaijmakers et al., 2010). Some lipopeptides were proven to confer protection against predation by protozoa. For example, massetolide and viscosin are involved in the protection of *Pseudomonas fluorescens* strains against the amoeba *Naegleria americana* causing lysis of the predator (Mazzola et al., 2009). Biosurfactants can change surface viscosity thereby influencing cell differentiation and motility giving advantage to surface colonization (Raaijmakers et al., 2010). Some of these molecules also play an important role in surface attachment and biofilm formation. Indeed, the disruption of *arfB*, involved in arthrofactin production, decreases swarming activity, and enhances biofilm formation (Roongsawang et al., 2003). Moreover, the chelation of metal ions has been described for several lipopeptides produced by *Bacillus* species (Raaijmakers et al., 2010). Regarding the antimicrobial activity of milkisin, this lipopeptide most probably confers another advantage than antagonism to the strain. Therefore, it would be interesting in the future to evaluate its role against predation or colonization and biofilm formation.

The analysis of the single-gene sequence indicates that *Pseudomonas* sp. UCMA 17988 belongs to a putative new species of the *P. koreensis* subgroup. However, in order to confirm this hypothesis, comparative bioinformatics analyses of whole genomes of *P. koreensis* subgroup species will have to be performed in the future (Mulet et al., 2010). Moreover, in further analyses, the description of this putative new species would also have to be detailed (e.g., phenotypic characteristics, cellular fatty acid composition). Among the *P. koreensis* subgroup, five species have been described to date: *P. koreensis*, *P. moraviensis*, *P. helmanticensis*, and *P. granadensis* species (described in soil samples all around the world) and *baetica* as a marine fish pathogen (Mulet et al., 2010; López et al., 2012; Ramírez-Bahena et al., 2014). Only three isolates of *P. koreensis* subgroup, isolated from water collected in a Mexican oasis, were found to produce biosurfactants. This production confers antagonistic activity toward *B. subtilis* and *Exiguobacterium aurantiacum* but not *Enterococcus faecalis*, *S. aureus*, *E. coli*, *Salmonella* Typhi, and *Bacillus liquefaciens* strains. However, as far as we know, these biosurfactants have never been characterized (Toribio et al., 2011).

Interestingly, milkisin is involved in the antagonistic effect of the strain against a Gram-negative bacterial strain (*Salmonella* Newport CIP 105629) thus conferring an atypical activity to the *Pseudomonas* sp. UCMA 17988 strain. Gram-negative bacteria are very common in dairy foods where they can reach a high level. Most of them are regularly considered as indicators of poor hygiene or may constitute a health risk if pathogenic. Nevertheless, some of them may also positively contribute to the quality of dairy products (Quigley et al., 2013). As already mentioned, *Pseudomonas* genus is mostly known for its spoilage potential. However, some strains producing such antimicrobial lipopeptides may contribute to the antimicrobial barrier against pathogens such as *Salmonella* or *S. aureus* in raw milk. In perspective, it might be interesting to characterize the spoilage potential of the strain *Pseudomonas* sp. UCMA 17988 in the near future and to search for new lipopeptide producers in raw milk.

*Pseudomonas* species are also associated to plant phyllosphere and rhizosphere and can be found in the dominant microbiota during the whole shelf life of leafy greens (Dees et al., 2015) and pre-prepared vegetables (Jackson et al., 2013). Because *Salmonella* is an important cause of foodborne outbreaks (Callejón et al., 2015) associated to the consumption of vegetables, the presence of such *Pseudomonas* strains as a protective culture might be of interest and would deserve to be investigated.

## Author Contributions

MSc performed the laboratory experiments, analyzed the data, and written up the manuscript. JG contributed to laboratory experiments and data analysis. HO and MSe performed the NMR experiments and analyzed the data. BB performed the mass spectrometry analysis. DG and VS performed the antifungal analysis. ND supervised the entire study. All authors proofread and reviewed the manuscript.

## Conflict of Interest Statement

The authors declare that the research was conducted in the absence of any commercial or financial relationships that could be construed as a potential conflict of interest.

## Footnotes

1 http://bioinfo.lifl.fr/norine/
