# Corrigendum: Characterization of Milkisin, a Novel Lipopeptide With Antimicrobial Properties Produced By *Pseudomonas* sp. UCMA 17988 Isolated From Bovine Raw Milk

**DOI:** 10.3389/fmicb.2020.01323

**Published:** 2020-06-29

**Authors:** Margot Schlusselhuber, Justine Godard, Muriel Sebban, Benoit Bernay, David Garon, Virginie Seguin, Hassan Oulyadi, Nathalie Desmasures

**Affiliations:** ^1^UNICAEN, UNIROUEN, ABTE, Normandie Université, Caen, France; ^2^UNIROUEN, INSA Rouen, CNRS, COBRA, Normandie Université, Rouen, France; ^3^UNICAEN, SF ICORE 4206, Normandie Université, Caen, France

**Keywords:** antimicrobial activity, *Pseudomonas*, milkisin, amphisin, lipopeptide

In the original article, there was a mistake in Figure 4, Figure 5, Figure 6, Figure 8, and Supplementary Table S1 as published. Indeed, the quasi–identical positions of different amino acids signals in NMR spectra (Murphy's Law), lead to a mis-interpretation of the sequence of amino acids of milkisin that has to be corrected. Thus, NMR spectra figures and Supplementary Table S1 were corrected as well as Figures 4 and 8. The corrected figures and Supplementary Table S1 appear below.

**Figure 4:**
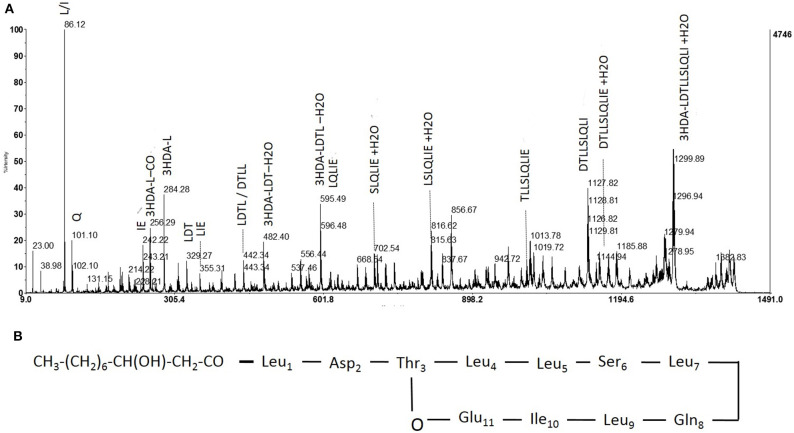
Fragmentation pattern of lipopeptide *m/z* 1409 and proposed structure. **(A)** Product ions obtained by fragmentation using MALDI-TOF. **(B)** Determined structure of purified lipopeptide (*m/z* 1409) based on mass spectrometric and NMR analysis.

**Figure 5:**
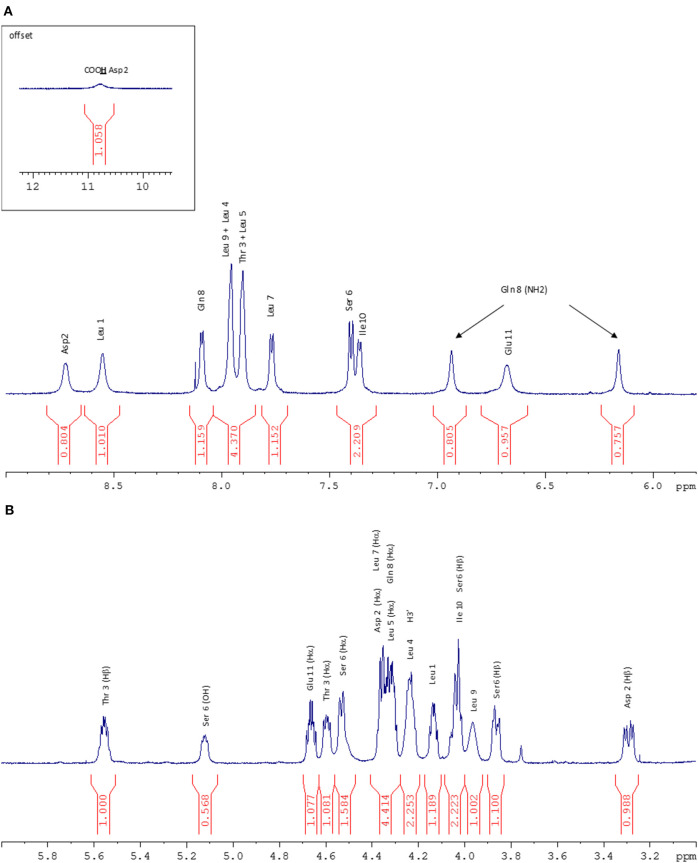
**(A)** NH zone of the ^1^H NMR spectrum (600 MHz, 298 K, Acetone-d_6_). **(B)** Hα zone of the ^1^H NMR spectrum (600 MHz, 298 K, Acetone-d6).

**Figure 6:**
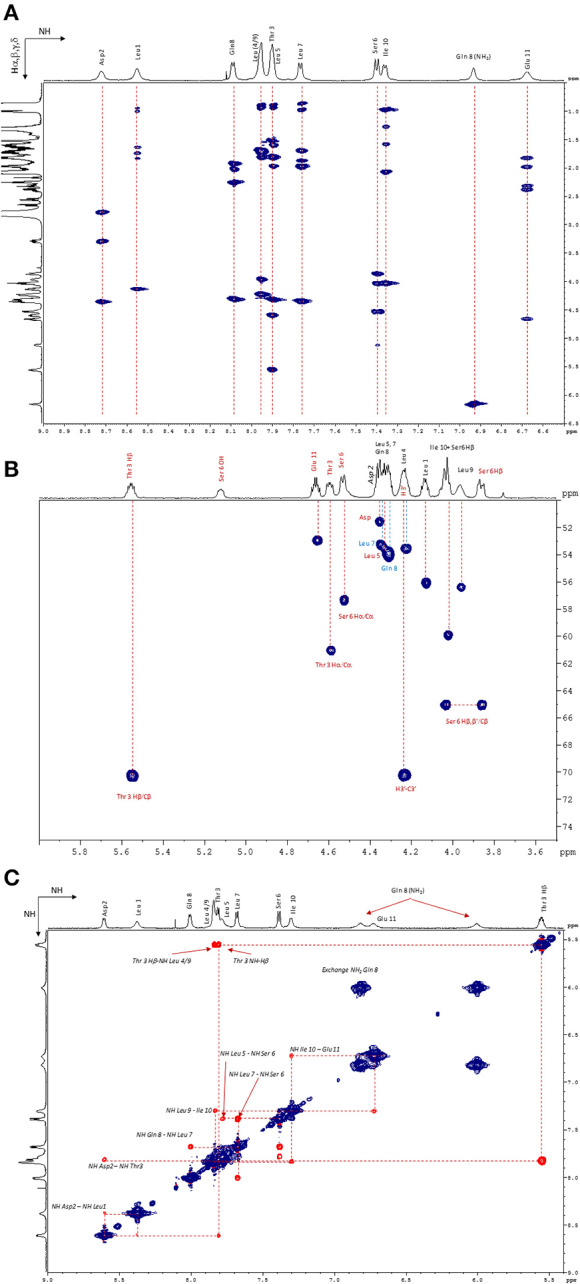
**(A)** NH/Hα, β, and γ zone of the ^1^H−1H TOCSY NMR spectrum (600 MHz, 298 K, Acetone-d_6_). **(B)** Hα/Cα zone of the ^1^H–^13^C HSQC NMR spectrum (600 MHz, 298 K, Acetone-d_6_). **(C)** NH/NH zone of the ^1^H–^1^H NOESY NMR spectrum (600 MHz, 323 K, Acetone-d6).

**Figure 8:**
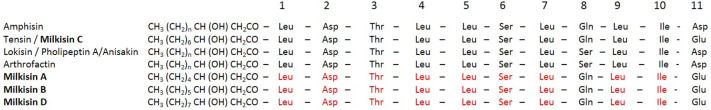
Alignment of lipopeptide milkisin isoforms produced by *Pseudomonas* sp. UCMA 17988 with members of amphisin group.

**Table S1:** ^1^H (600 MHz) and ^13^C NMR (150 MHz) data measured at 298 K in acetone-d6.

**Residue**	**Proton**	**δ (^**1**^H, ppm) (multiplicity, integration), J(Hz)**	**Carbon**	**δ (^**13**^C, ppm)**
Leu^1^	NH	8.55 (bs, 1H)	–	–
	α	4.13 (dt, 1H), ^3^J_NH−α_ = 7.9; ^3^J_α-β/β′_ = 4.8	α	56.12
	β	1,74 (m, 7H)	β	*40.50*
	β′	1,65 (m, 6H)		
	γ	1.82 (m, 7H)	γ	*25.20*
	δ/δ′	0.98 (m, 45H)	δ/δ′	23.87 (or 15.90)
			C=O	*ND*
Asp^2^	NH	8.73 (bs, 1H)		
	α	4.36 (m, 4H)	α	51,58 or 53,31
	β	3.29 (dd, 1H), ^2^J_β-β′_ = 16.62; ^3^J_α−β_ = 6.84 Hz	β	34.28
	β′	2.78 (with water)		
			C=O/COOH	173.63; 172.04
Thr^3^	NH	7,91 (bs,2H)	–	–
	α	4,59 (dd,1H), ^3^J_NH−α_ = 5,9; ^3^J_α−β_ = 10,2 Hz	α	61.11
	β	5,56 (dq,1H), ^3^J_α−β_ = 10,2; ^3^J_β−γ_ = 6,1 Hz	β	70.33
	γ	1.52 (d, 4H) with H5′, ^3^J_β−γ_ = 6,1 Hz	γ	17.73
			C=O	–
Leu^4^	NH	7.96 (bs, 2H)		
	α	4.24 (m, 2H)	α	53.57
	β	*1.69 (m, 7H)*	β	*40.8*
	β′	*1.65 (m, 6H)*		
	γ	ND	γ	
	δ/δ′	0.92 (m, 45H)	δ/δ′	
			C=O	
Ile^10^	NH	7.36 (d, 1H), ^3^J_NH−α_ = 5,9	–	–
	α	4,03 (m,2H)	α	60.04
	β	2.07 (with solvent)	β	36.32
	γ	1.59 (m, 6H)	γ	26.03
	γ'	1.27 (m, 13H)		
	δ_CH3_/γ_CH3_	0.97 (m, 45H)	δ	23.87 or 15.90
			C=O	
Glu^11^	NH	6.68	–	
	α	4.66 (dt, 1H), ^3^J_NH−α_ = 5,3; ^3^J_α-β/β′_ = 8,9	α	53.02
	β	1.97	β	29.49 (or 24.97)
	β′	1.83		
	γ	2.38	γ	30.18
	γ′	2.32		
			C=O	*172.93*
Leu^5^	NH	7,91 (bs,2H)	–	–
	α	4.32 (m, 4H)	α	54.03
	β	*1.96*	β/β′	*25.0*
	β′	*1.86*		
	γ	*1.79*	γ	
	δ/δ′	*0.90*	δ/δ′	22.60 or 11.16
			C=O	*173.71*
Ser^6^	NH	7,40 (d,1H), ^3^J_NH−α_ = 8,9	–	–
	α	4,52 bdt,1H, ^3^J_NH−α_ = 8,9; ^3^J_α-β/β′_ = 2,40	α	57.39
	β	4,03 (m,2H)	β	65.10
	β′	3.87 (dd, 1H), ^2^J_β-β′_ = 11.7; ^3^J_β′-OH_ = 4.8		
	OH	5.12 (dd, 1H), ^3^J_β′-OH_ = 4.8; ^3^J_β−*OH*_ = 8.9		
			C=O	*173.11*
Leu^7^	NH	7,77 (d,1H), ^3^J_NH−α_ = 7.6	–	–
	α	4.35 (m, 4H)	α	54.03
	β	*1.99*	β	
	β′	*1.90*		
	γ	1.69	γ	
	δ	0.96	δ	24, 00 or 15.82
	δ′	0.88		20.65 or 14.11
			C=O	
Gln^8^	NH	8.09 (d, 1H), ^3^J_NH−α_ = 6.7	–	–
	α	4.32 (m, 4H)	α	54.03
	β/β′	*1.95 or 2.00/1.92 (m, 6H)*	β/β′	*28.63*
	γ	2.25 (t, 2H), ^3^J_β−γ_ = 7.02	γ	32.24
	NH_2_	6.93 (bs, 1H)		
		6.16 (bs, 1H)		
			C=O	*173.42*
Leu^9^	NH	7,96 (bs, 2H)	–	–
	α	3.97 (bs, 1H)	α	56.42
	β	*1.78 (m, 7H)*	β	*40.81*
	β′	1.74 (m,7H)		
	γ	ND	γ	
	δ/δ′	0.93	δ/δ′	
			C=O	
3HDA	2[	2.66 (dd, 2H) ^2^J_2′a-2′b_ = 13.91; ^3^J_2′a-3′_ = 3.99	2′	43.87
		2.55 (dd, 2H) ^2^J_2′a-2′b_> = 13.91; ^3^J_2′b-3′_ = 10.40		
	3′	4.24	3′	70.33
	4′	*1.88–1.24*	4′	
	5′	1.52 (d, 4H) / 1.41	5′	26.14
	6′	*1.88–1.24*	6′	
	7′	*1.88–1.24*	7′	
	8′	*1.88–1.24*	8′	
	9′	*1.88–1.24*	9′	
	10′	0.82–1.00	10′	
			C=O	

*bs, broad signal; m, multiplet; d, doublet; t, triplet; q, quadruplet; ND, Not determined. In italic, the uncertain values*.

In the original article, there was error. As previously mentioned, the quasi–identical positions of different amino acids signals in NMR spectra (Murphy's Law), lead to a mis-interpretation of the sequence of amino acids of milkisin that has to be corrected. The corrections have been made to the **Abstract** and the **Results** section, Extraction and Structural Analysis of Biosurfactants sub-section respectively:

“Biosurfactants such as lipopeptides are amphiphilic compounds produced by microorganisms such as bacteria of the genera of *Pseudomonas* and *Bacillus*. Some of these molecules proved to have interesting antimicrobial, antiviral, insecticide and/or tensio-active properties that are potentially useful for the agricultural, chemical, food, and pharmaceutical industries. Raw milk provides a physicochemical environment that is favorable to the multiplication of a broad spectrum of microorganisms. Among them, psychrotrophic bacterial species, especially members of the genus *Pseudomonas*, are predominant and colonize milk during cold storage and/or processing. We isolated the strain *Pseudomonas* sp. UCMA 17988 from raw cow milk, with antagonistic activity against *Listeria monocytogenes, Staphylococcus aureus*, and *Salmonella enterica* Newport. Antimicrobial molecules involved in the antagonistic activity of this strain were characterized. A mass spectrometry analysis highlighted the presence of four lipopeptides isoforms. The major isoform (1409 *m/z*), composed of 10 carbons in the lipidic chain, was named milkisin C. The three other isoforms detected at 1381, 1395, and 1423 *m/*z, that are concomitantly produced, were named milkisin A, B and D, respectively. The structure of milkisin, as confirmed by NMR analyses, is closely related to amphisin family. Indeed, the peptidic chain was composed of 11 amino acids, 9 of which are conserved among the family. In conclusion, *Pseudomonas* sp. UCMA 17988 produces new members of the amphisin family which are responsible for the antagonistic activity of this strain.”

“Hereafter, collected fractions were analyzed by MALDI-TOF mass spectrometry. The spectra also highlighted the presence of four molecules (Figure 4). Two intense pseudomolecular ions [M+H]+ at *m/z* 1409.49 and 1395.48 were observed. Corresponding sodium and potassium adducts were also found at *m/z* [M+Na]+ at 1417 and 1431.60 and *m/z* [M+K]+ at 1433.57 and 1447.57 respectively. The two other molecules could be detected through their potassium and/or sodium adducts (*m/z* [M+Na]+ at 1403; *m/z* [M+K]+ at 1419.57 and 1461.58). The presence of four molecules harboring a difference of 14 Da was consistent with the presence of lipopeptides isoforms. Indeed, such difference is typical of the addition or substitution of a methyl group in the fatty acid chain. A MS/MS analysis was performed on the dominant [M+H]+ ion (*m/z* at 1409.49). The fragmentation spectrum obtained is shown in Figure 5. Fragments were assigned by subtraction of the different peaks between them. The ions with 284 Da and 86 Da masses were observed and respectively correspond to fragment 3-hydroxy-fatty acid-Leu/Ile and immonium ion of leucine or isoleucine. The following linear sequence was proposed: 3HDA-Leu/Ile1-Asp2-Thr3-Leu/Ile4-Leu/Ile5-Ser6-Leu/Ile7-Gln8-Leu/Ile9-Leu/Ile10-Glu11 with cyclisation between Thr3 and Glu11.”

The authors apologize for these errors and state that this does not change the scientific conclusions of the article in any way. The original article has been updated.

