# Supplementary material for: Characterization of Milkisin, a Novel Lipopeptide With Antimicrobial Properties Produced By Pseudomonas sp. UCMA 17988 Isolated From Bovine Raw Milk
Source: Front Microbiol. 2018 May 28;9:1030. doi: 10.3389/fmicb.2018.01030 (PMC5985324; doi:10.3389/fmicb.2018.01030)
Supplement: Supplementary file 1 [file Table_1.docx]

**Table 1**: ^1^H (600MHz) and ^13^C NMR (150MHz) data measured at 298 K in acetone-d6. bs: broad signal, m: multiplet, d: doublet, t: triplet, q: quadruplet. ND: Not determined. In italic, the uncertain values.

| Residue | Proton | δ (^1^H, ppm) (multiplicity, integration), J(Hz) | Carbon | δ (^13^C, ppm) |
| --- | --- | --- | --- | --- |
| Leu^1^ | NH | 8.55 (bs, 1H) | - | - |
|  | α | 4.13 (dt, 1H),  ^3^J_NH-α_ = 7.9 ;  ^3^J_α-β/β’_ = 4.8 | α | 56.12 |
|  | β | 1,74 (m, 7H) | β | *40.50* |
|  | β’ | 1,65 (m, 6H) |  |  |
|  | γ | 1.82 (m, 7H) | γ | *25.20* |
|  | δ/δ’ | 0.98 (m, 45H) | δ/δ’ | 23.87 (or 15.90) |
|  |  |  | C=O | *ND* |
| Asp^2^ | NH | 8.73 (bs, 1H) |  |  |
|  | α | 4.36 (m, 4H) | α | 51,58 or 53,31 |
|  | β | 3.29 (dd, 1H),  ^2^J_β-β’_= 16.62 ;  ^3^J_α-β_= 6.84 Hz | β | 34.28 |
|  | β’ | 2.78 (with water) |  |  |
|  |  |  | C=O/COOH | 173.63 ; 172.04 |
| Thr^3^ | NH | 7,91 (bs,2H) | - | - |
|  | α | 4,59 (dd,1H),  ^3^J_NH-α_= 5,9 ;  ^3^J_α-β_= 10,2 Hz | α | 61.11 |
|  | β | 5,56 (dq,1H),  ^3^J_α-β_ = 10,2 ;  ^3^J_β-γ_ = 6,1 Hz | β | 70.33 |
|  | γ | 1.52 (d, 4H) with H5’,  ^3^J_β-γ_ = 6,1 Hz | γ | 17.73 |
|  |  |  | C=O | - |
| Leu^4^ | NH | 7.96 (bs, 2H) |  |  |
|  | α | 4.24 (m, 2H) | α | 53.57 |
|  | β | *1.69 (m, 7H)* | β | *40.8* |
|  | β’ | *1.65 (m, 6H)* |  |  |
|  | γ | ND | γ |  |
|  | δ/δ’ | 0.92 (m, 45H) | δ/δ’ |  |
|  |  |  | C=O |  |
| Ile^10^ | NH | 7.36 (d, 1H),  ^3^J_NH-α_= 5,9 | - | - |
|  | α | 4,03 (m,2H) | α | 60.04 |
|  | β | 2.07 (with solvent) | β | 36.32 |
|  | γ | 1.59 (m, 6H) | γ | 26.03 |
|  | γ’ | 1.27 (m, 13H) |  |  |
|  | δ_CH3_/γ_CH3_ | 0.97 (m, 45H) | δ | 23.87 or 15.90 |
|  |  |  | C=O |  |
| Glu^11^ | NH | 6.68 | - |  |
|  | α | 4.66 (dt, 1H),  ^3^J_NH-α_= 5,3 ;  ^3^J_α-β/β’_= 8,9 | α | 53.02 |
|  | β | 1.97 | β | 29.49 (or 24.97) |
|  | β’ | 1.83 |  |  |
|  | γ | 2.38 | γ | 30.18 |
|  | γ’ | 2.32 |  |  |
|  |  |  | C=O | *172.93* |
| Leu^5^ | NH | 7,91 (bs,2H) | - | - |
|  | α | 4.32 (m, 4H) | α | 54.03 |
|  | β | *1.96* | β/β’ | *25.0* |
|  | β’ | *1.86* |  |  |
|  | γ | *1.79* | γ |  |
|  | δ/δ’ | *0.90* | δ/δ’ | 22.60 or 11.16 |
|  |  |  | C=O | *173.71* |
| Ser^6^ | NH | 7,40 (d,1H),  ^3^J_NH-α_ = 8,9 | - | - |
|  | α | 4,52 (bdt,1H,  ^3^J_NH-α_ = 8,9 ;  ^3^J_α-β/β’_ = 2,40 | α | 57.39 |
|  | β | 4,03 (m,2H) | β | 65.10 |
|  | β’ | 3.87 (dd, 1H),  ^2^J_β-β’_ = 11.7 ;  ^3^J_β’-OH_ = 4.8 |  |  |
|  | OH | 5.12 (dd, 1H),  ^3^J_β’-OH_ = 4.8 ;  ^3^J_β-OH_ = 8.9 |  |  |
|  |  |  | C=O | *173.11* |
| Leu^7^ | NH | 7,77 (d,1H),  ^3^J_NH-α_ = 7.6 | - | - |
|  | α | 4.35 (m, 4H) | α | 54.03 |
|  | β | *1.99* | β |  |
|  | β’ | *1.90* |  |  |
|  | γ | 1.69 | γ |  |
|  | δ | 0.96 | δ | 24, 00 or 15.82 |
|  | δ’ | 0.88 |  | 20.65 or 14.11 |
|  |  |  | C=O |  |
| Gln^8^ | NH | 8.09 (d, 1H),  ^3^J_NH-α_ = 6.7 | - | - |
|  | α | 4.32 (m, 4H) | α | 54.03 |
|  | β/β’ | *1.95 or 2.00/1.92 (m, 6H)* | β/β’ | *28.63* |
|  | γ | 2.25 (t, 2H),  ^3^J_β-γ_ = 7.02 | γ | 32.24 |
|  | NH_2_ | 6.93 (bs, 1H) |  |  |
|  |  | 6.16 (bs, 1H) |  |  |
|  |  |  | C=O | *173.42* |
| Leu^9^ | NH | 7,96 (bs, 2H) | - | - |
|  | α | 3.97 (bs, 1H) | α | 56.42 |
|  | β | *1.78 (m, 7H)* | β | *40.81* |
|  | β’ | 1.74 (m,7H) |  |  |
|  | γ | ND | γ |  |
|  | δ/δ’ | 0.93 | δ/δ’ |  |
|  |  |  | C=O |  |
| 3HDA | 2’ | 2.66 (dd, 2H)  ^2^J_2’a-2’b_ = 13.91 ;  ^3^J_2’a-3’_ = 3.99 | 2’ | 43.87 |
|  |  | 2.55 (dd, 2H)  ^2^J_2’a-2’b_ = 13.91 ;  ^3^J_2’b-3’_ = 10.40 |  |  |
|  | 3’ | 4.24 | 3’ | 70.33 |
|  | 4’ | *1.88-1.24* | 4’ |  |
|  | 5’ | 1.52 (d, 4H) / 1.41 | 5’ | 26.14 |
|  | 6’ | *1.88-1.24* | 6’ |  |
|  | 7’ | *1.88-1.24* | 7’ |  |
|  | 8’ | *1.88-1.24* | 8’ |  |
|  | 9’ | *1.88-1.24* | 9’ |  |
|  | 10’ | 0.82-1.00 | 10’ |  |
|  |  |  | C=O |  |
