# Supplementary material for: Characterization of Milkisin, a Novel Lipopeptide With Antimicrobial Properties Produced By Pseudomonas sp. UCMA 17988 Isolated From Bovine Raw Milk
Source: Front Microbiol. 2018 May 28;9:1030. doi: 10.3389/fmicb.2018.01030 (PMC5985324; doi:10.3389/fmicb.2018.01030)
Supplement: Supplementary file 2 [file Presentation_1.pdf]

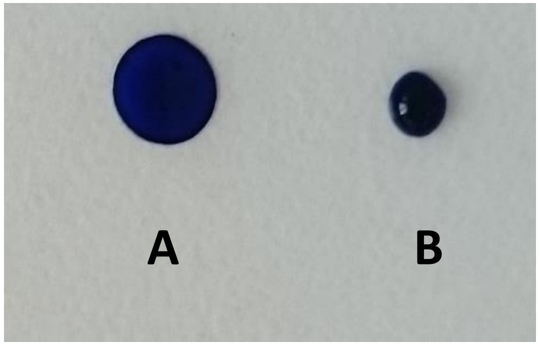

Figure S1. Biosurfactant production screening by drop-collapse method. The cell free supernatant of *Pseudomonas* sp. UCMA 17988 (A) was compared to sterile MSM medium as control (B).

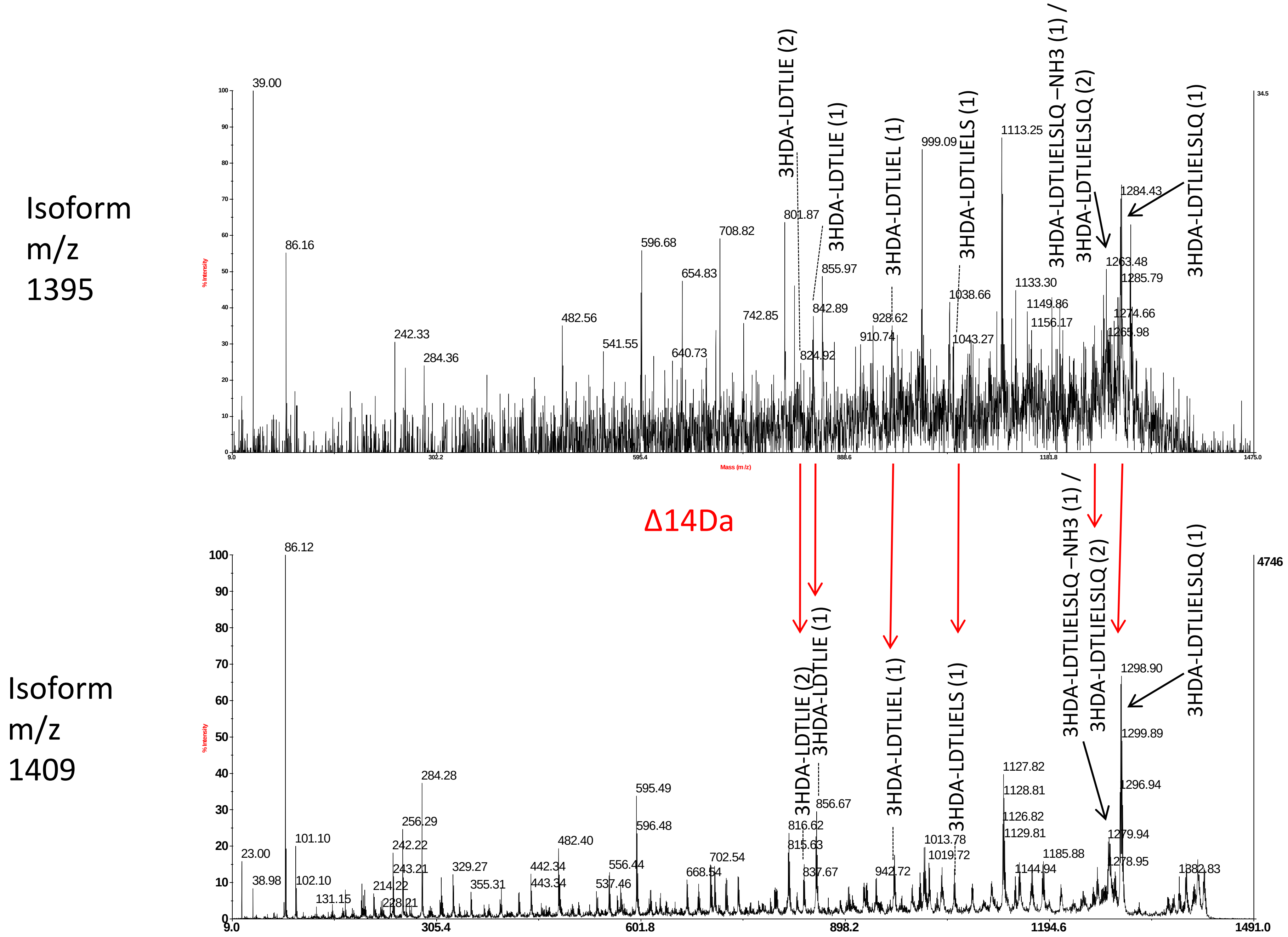

Figure S2. Comparison of fragmentation patterns of isoforms m/z 1395 and 1409 ([M+H]+). A difference of 14 Daltons between fragments containing the lipid chain is highlighted by red arrows.

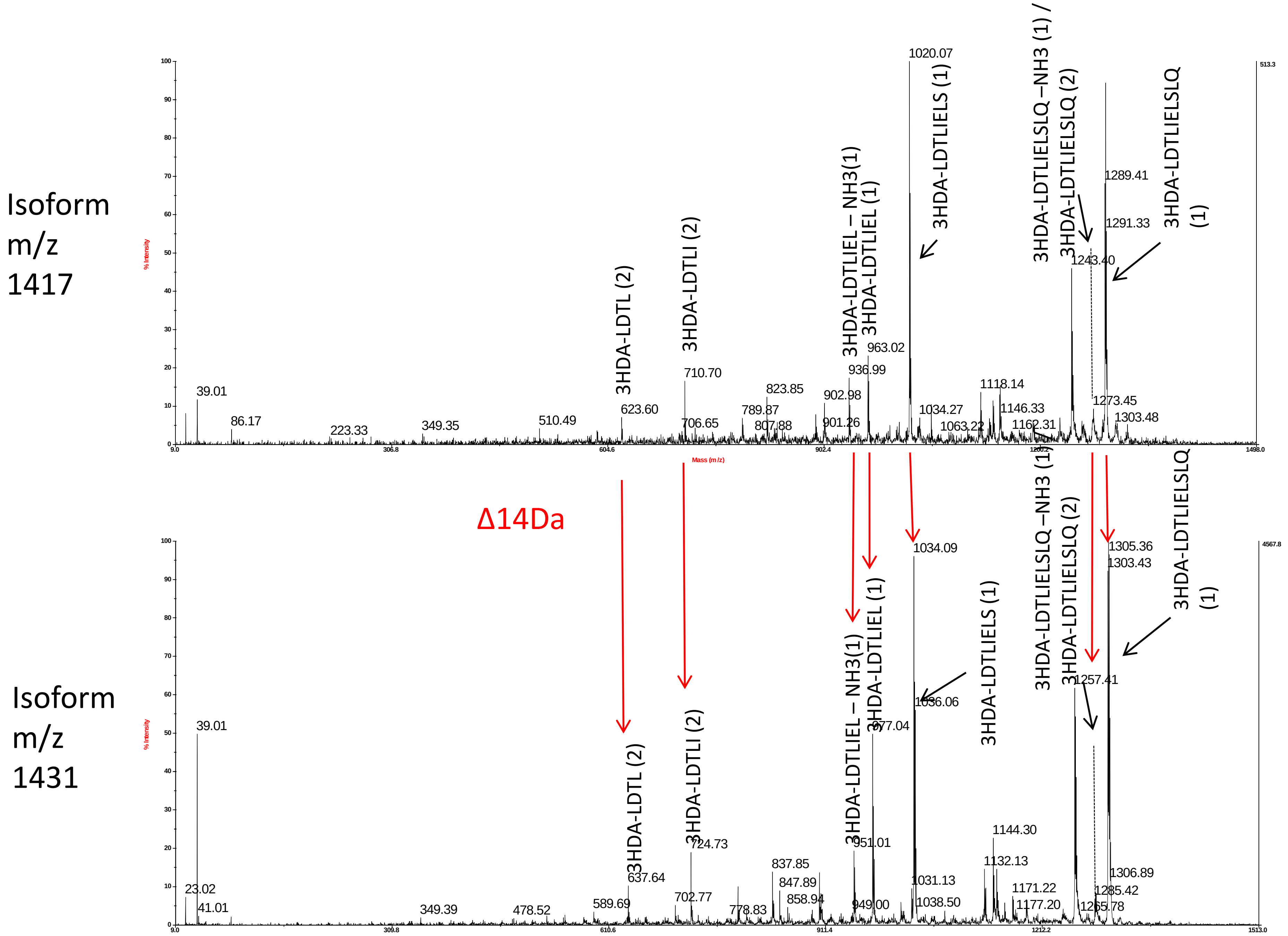

Figure S3. Comparison of fragmentation patterns of isoforms m/z 1417 and 1431 ([M+Na]+). A difference of 14 Daltons between **f**ragments containing the lipid chain is highlighted by arrows.

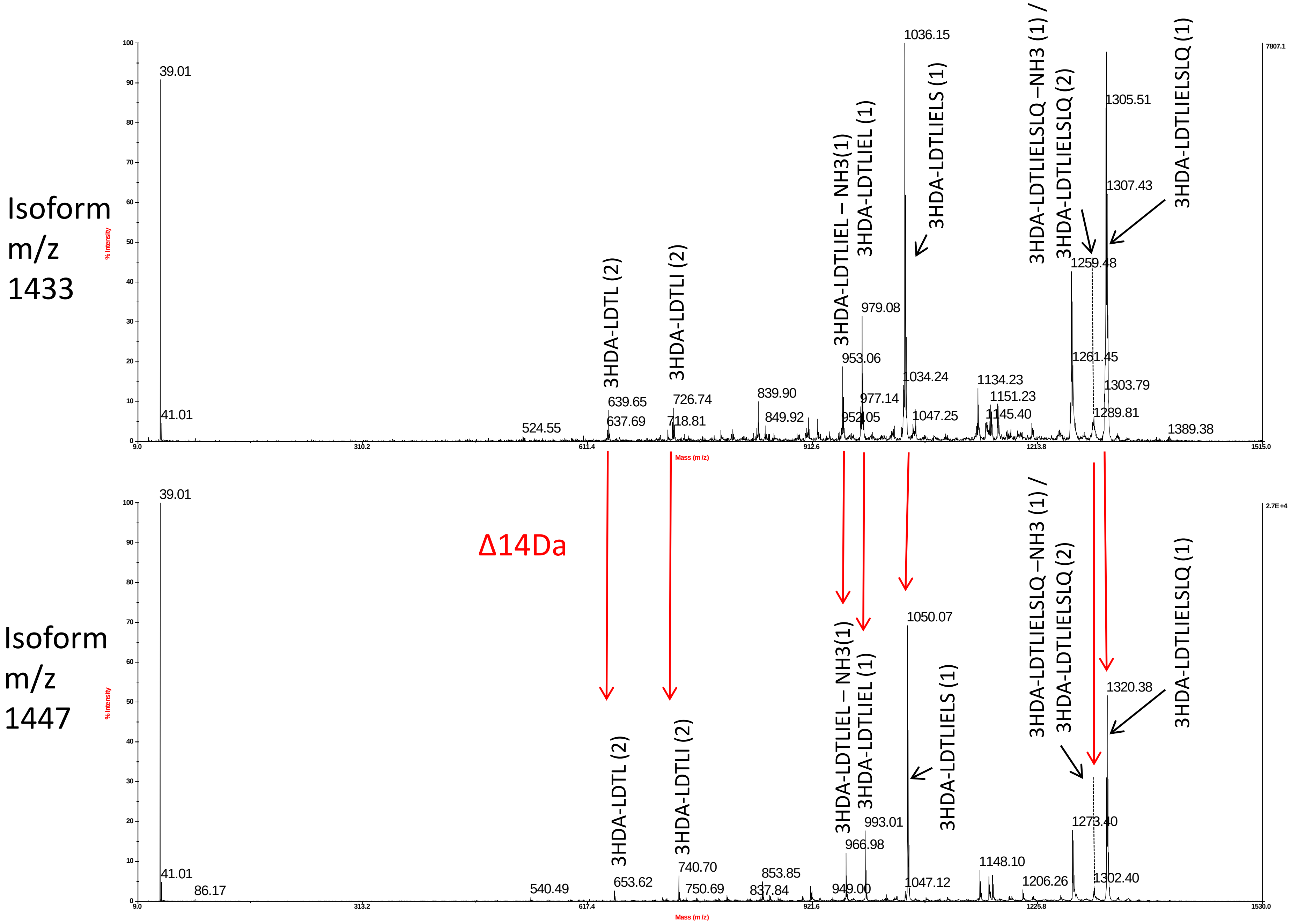

Figure S4. Comparison of fragmentation patterns of isoforms m/z 1433 and 1447 ([M+K]+). A difference of 14 Daltons between **f**ragments containing the lipid chain is highlighted by arrows.
